# Effect of atorvastatin treatment on circulating adiponectin: a meta-analysis of randomized controlled trials

**DOI:** 10.1186/s12944-019-1172-7

**Published:** 2019-12-23

**Authors:** Xiaoyu Liu, Wei Zhang, Ming Zhao, Guowei Jia, Rongguo Sun

**Affiliations:** The Third Department of Cardiology, Cangzhou Central Hospital, No. 201 Xinhuazhong Road, Yunhe District, Cangzhou, 061000 China

**Keywords:** Atorvastatin, Adiponectin, Meta-analysis, Randomized controlled trials

## Abstract

**Background:**

Influences of atorvastatin on atherosclerosis and glycemic metabolism may be related to its potential impact on circulating adiponectin, an adipocyte that exerts anti-inflammatory, ant-atherosclerotic, and anti-oxidative effects. However, results of previous randomized controlled trials (RCTs) were not consistent. We performed a meta-analysis of RCTs to systematic evaluate the influence of atorvastatin on circulating adiponectin.

**Methods:**

Relevant studies were identified via search of electronic databases of PubMed, Embase, and Cochrane’s Library. A random-effect model was applied to pool the results via incorporating the potential heterogeneity. Predefined meta-regression and subgroup analyses were used to evaluate the influences of study characteristics on the outcome.

**Results:**

Fourteen datasets from ten RCTs including 931 patients were included. Pooled results showed that atorvastatin did not significantly affect circulating adiponectin as compared with controls (weighed mean difference = − 0.27 μg/mL, 95% confidence interval: − 0.89 to 0.35 μg/mL, *p* = 0.39). Results of univariate meta-regression analyses showed that study characteristics including number of patients, mean age, proportion of male patients, body mass index, dose of atorvastatin, or treatment duration did not significantly affect the outcome (p all > 0.05). Moreover, subgroup analyses showed that atorvastatin did not significantly affect circulating adiponectin in studies stratified according to these study characteristics (p all > 0.05).

**Conclusions:**

Atorvastatin treatment does not significantly affect circulating adiponectin. Influences of atorvastatin on atherosclerosis and glycemic metabolism are not likely to be mediated by modulation of circulating adiponectin.

## Background

Atorvastatin is one of the most commonly prescribed statins, the efficacies of which for the prevention and treatment of cardiovascular diseases (CVDs), including coronary artery diseases [1], stroke [2], and atrial fibrillation [3] have been well documented. As a high-potential pleiotropic statin, atorvastatin exerts various pharmacological efficacies besides lowering of low-density lipoprotein cholesterol, such as anti-inflammation, anti-oxidative stress related injuries, and anti-thrombosis [4, 5]. Moreover, recent evidence suggests that atorvastatin treatment may cause worsening of glycemic control in patients with diabetes mellitus (DM) and even lead to increased risk of new-onset DM in general population [6]. However, the mechanisms underlying the potential harmful influences of atorvastatin on glycemic metabolism remain unclear.

Adiponectin is an anti-inflammatory cytokine secreted from adipocytes. Accumulating evidence suggests that adiponectin may be a protective factor for atherosclerosis via its inhibitory effects on inflammation, oxidation, platelet aggregation, and thrombosis formation [7]. Epidemiological studies also indicate that higher adiponectin may be a marker of lower risk for CVDs [8], although the results were not consistent [9, 10]. In addition, adiponectin has also been indicated to favorably affect glycemic metabolism via maintaining the sensitivity of insulin, and higher circulating level of adiponectin has been related with lower risk of diabetes [11, 12]. Previous studies demonstrated that the cardiovascular and metabolic influences of statins may be related to their effects on adiponectin [13, 14]. In a previous meta-analysis, statins were shown to increase the level of adiponectin [15]. However, subgroup analyses also showed that the influences of statins on circulating adiponectin were drug-specific [15]. Moreover, regimens of statin treatment and characteristics of the patients may also modify the influences of statin on adiponectin. A previous meta-analysis showed that simvastatin increases circulating adiponectin if administered for more than 12 weeks [16]. Another meta-analysis showed that pravastatin may increase adiponectin in males, but not in females [17]. As for the influences of atorvastatin on adiponectin, results of previous randomized controlled trials (RCTs) are not consistent [18–27]. An early RCT showed that atorvastatin 20 mg/d treated for 12 weeks was associated with significantly decreased circulating adiponectin in DM patients [18], while another study showed that treatment with atorvastatin 40 mg/d for 24 weeks increased adiponectin in patients with hyperlipidemia [25]. Therefore, we performed a meta-analysis to systematically evaluate the influence of atorvastatin on circulating adiponectin. We aimed to explore whether regimens of atorvastatin treatment or the characteristics of the patients may affect the outcome.

## Methods

### Database search

The PRISMA (Preferred Reporting Items for Systematic Reviews and Meta-Analyses) [28] and the Cochrane Handbook for Systematic Review [29] guidelines were followed through the design and performing of the meta-analysis. Electronic databases including PubMed, Embase, and the Cochrane’s Library were systematically searched for relevant studies using terms of “atorvastatin” combined with “adiponectin”. The searched was limited to human studies published in English. The final database search was performed on June 17, 2019. We also performed a manual screening of the related original articles and reviews for potential studies.

### Study selection

Studies were included if they met the following criteria: (1) published as full-length articles in English; (2) designed as RCTs; (3) included participants who were randomly allocated to a treatment group of oral atorvastatin and a control group of placebo or blank treatment; (4) with a treatment and follow-up duration of at least 1 week; and (5) reported the difference of the changes of circulating adiponectin from baseline between two groups as means and standard deviations (SDs). Review articles, editorials, preclinical studies, and non-RCT studies were excluded.

### Data extraction and quality evaluation

Two authors performed database search, study identification, data extraction, and quality assessment independently according to the predefined criteria. Discrepancies were solved by consensus with the corresponding author. For studies with multiple interventional groups (e.g. with multiple doses of atorvastatin), multiple datasets were considered and the sample size of the control group was equally split to each datasets to avoid a unit of analysis error as indicated by the Cochrane Handbook for Systematic Review [29]. Data extracted included characteristics regarding study design, patient status (disease status, sample size, mean age, gender, mean body mass index [BMI]), atorvastatin treatment (dose and treatment duration), and methods for the measurement of circulating adiponectin. The Cochrane’s Risk of Bias Tool was applied to evaluate the quality of the included studies [29]. This tool judges the quality of RCTs via seven domains concerning the following aspects of the studies, including criteria concerning sequence generation, allocation concealment, participant and personnel blinding, outcome assessor blinding, incomplete outcome data, selective outcome reporting, and other potential threats to validity.

### Statistical analysis

We used the RevMan (Version 5.1; Cochrane Collaboration, Oxford, UK) and Stata software (version 12.0; Stata Corporation, College Station, TX, USA) for the meta-analysis and statistical analysis. Weighted mean difference (WMD) with 95% confidence intervals (CI) was used to present the outcome. Heterogeneity among the included studies was evaluated using Cochrane’s Q test, and significant heterogeneity was identified at *p* values < 0.10 [29]. We also calculated the I^2^ statistic to reflect the heterogeneity, which indicates the percentages of total variation across studies that are due to heterogeneity rather than chance. Significant heterogeneity was considered if I^2^ > 50% [30]. A random-effect model was used to pool the results since this model could incorporate the heterogeneity of the studies and therefore proved a more generalized result. The influences of the predefined study characteristics, including number of participants, age, gender, mean BMI, dose of atorvastatin and treatment duration, on the outcome were analyzed via univariate meta-regression and subgroup analyses. Medians of the continuous variables were used as cut-off values. Potential publication bias was assessed with a funnel plot and Egger’s regression asymmetry test [31]. *P* values were two-tailed and statistical significance was set at 0.05.

## Results

### Literature search and study identification

**Fig. 1:**
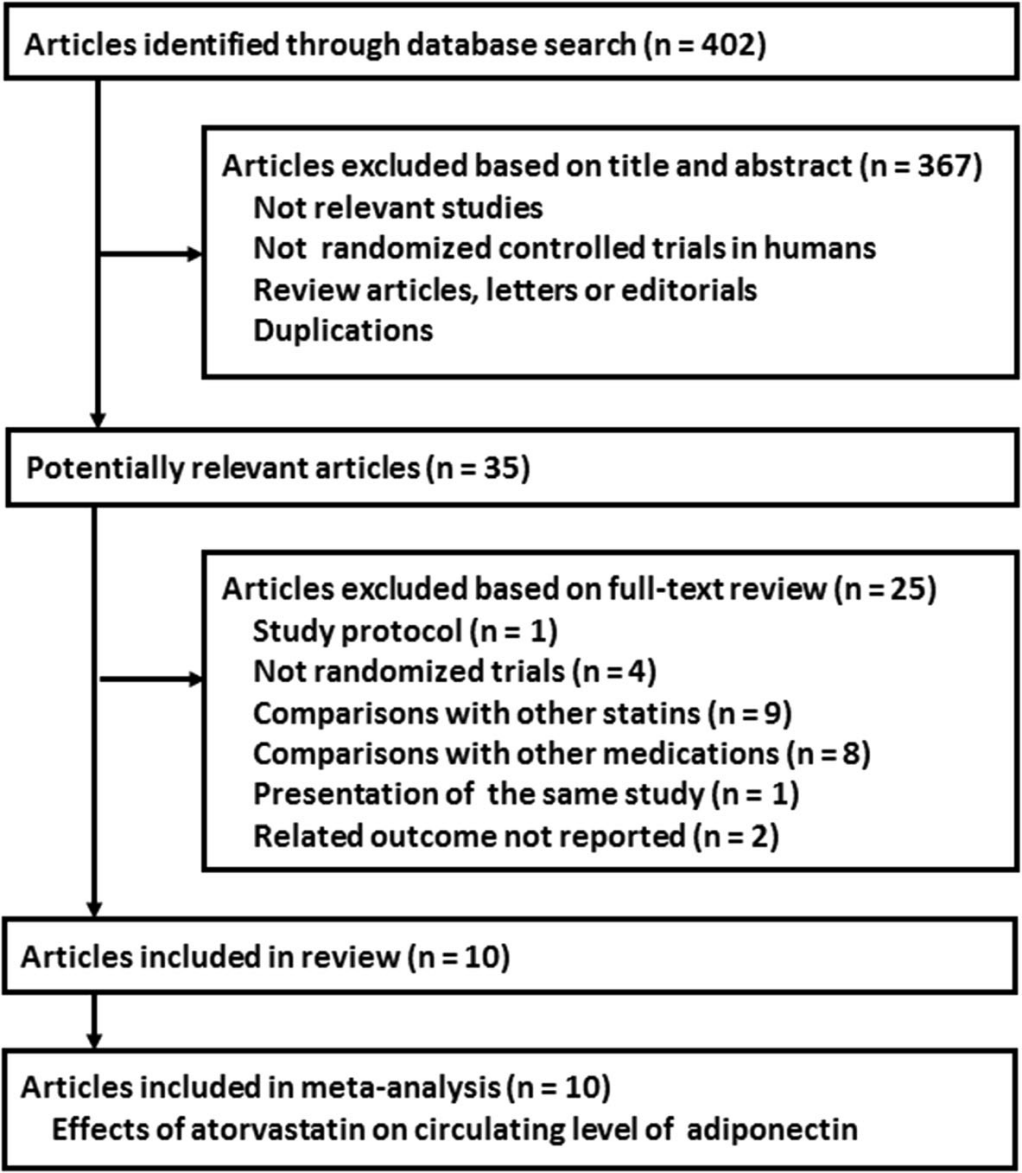
Flowchart of database search and study identification

The flowchart of database search was summarized in Fig. 1. Overall, 402 studies were identified after the initial database search, and 367 were excluded based on titles and abstracts mainly because they were irrelevant to the study purpose. The remaining 35 studies underwent full-text review, and 25 studies were further excluded because one of them was a study protocol, four were not RCTs, nine evaluated other statins rather than atorvastatin, eight compared atorvastatin with active medications rather than placebo or no treatment, one was repeated report of an included study, and the other two did not report outcomes regarding circulating adiponectin. Finally, ten RCTs were included in our meta-analysis [18–27].

### Study characteristics and quality evaluation

Since one study included two intervention arms of atorvastatin 10 and 80 mg/d, the other study included four intervention arms of atorvastatin 10, 20, 40, and 80 mg/d, these arms were included separately. Overall, our meta-analysis included ten RCTs [18–27] with 14 datasets. The characteristics of the included studies were presented in Table 1. Among the included studies, three were double-blinded and placebo-controlled RCTs [18, 19, 21]. Patients with various disease statuses were included, such as those with hyperlipidemia [19, 23, 24, 27], T2DM [18, 21, 22], hypertension [26], coronary artery disease [20], and rheumatic arthritis [25]. The sample sizes of the included comparisons varied from 30 to 109. The mean ages of the included patients ranged between 50.9 to 60.9 years, with varying proportions of male participants. Various doses of atorvastatin (10, 20, 40, 80 mg/d) were applied. The treatment durations varied from four to 30 weeks. In three studies, radioimmunoassay was used to measure circulating adiponectin [18, 20, 26], while for the other studies [19, 21–25, 27], enzyme-linked immunosorbent assay was applied. The details of quality assessment via the Cochrane’s Risk of bias tool were presented in Table 2. The quality of the included RCTs was generally modest, with total scores of one to four in the included studies.

**Table 1:** Characteristics of included studies

Author (year)	Design	Population	Number of subjects	Mean age	Male	Mean BMI	Dose	Follow-up durations	Adiponectin measurement
				years	%	Kg/m^2^	mg/d	weeks	
Shetty 2004	R, DB, PC	DM patients or subjects at risk for T2DM	77	51.1	55.8	29.2	20	12	RIA
Koh 2005	R, DB, PC, CO	Combined HL patients	56	56.0	41.1	25.5	10	8	ELISA
Chan 2008	R	Post-PCI CAD patients	60	65.0	71.7	25.5	10	24	RIA
von Eynatten 2009	R, SB, PC	T2DM patients	75	60.9	69.3	28.2	40	8	ELISA
van Hoek 2009–10 mg^a^	R, DB, PC	T2DM patients	109	59.3	55.4	30.7	10	30	ELISA
van Hoek 2009–80 mg^a^	R, DB, PC	T2DM patients	108	59.6	50.7	30.9	80	30	ELISA
Carnevale 2010	R, SB	HC patients	36	55.2	47.2	25.2	10	4	ELISA
Koh 2010–10 mg^b^	R, SB, PC	HC patients	53	55.6	51.2	24.8	10	8	ELISA
Koh 2010–20 mg^b^	R, SB, PC	HC patients	55	57.2	50.0	24.9	20	8	ELISA
Koh 2010–40 mg^b^	R, SB, PC	HC patients	54	58.0	51.7	25.0	40	8	ELISA
Koh 2010–80 mg^b^	R, SB, PC	HC patients	51	56.4	51.2	24.9	80	8	ELISA
Koh 2011	R, SB, PC, CO	HTN patients	42	53.0	52.4	25.5	20	8	RIA
El-Barbary 2011	R, OL	RA patients	30	54.2	16.7	25.7	40	24	ELISA
Buldak 2012	R, SB	HL patients with IFG	37	50.9	54.1	28.1	10	12	ELISA

*Abbreviations*: *BMI* body mass index, *R* random, *DB* double-blinded, *PC* placebo controlled, *CO* crossover, *SB* single-blinded, *OL* open label, *ELISA* enzyme-linked immunosorbent assay, *RIA* radioimmunoassay, *HC* hypercholesterolemic, *HL* hyperlipidemic, *HTN* hypertension, *T2DM* type 2 diabetes mellitus, *DM* diabetes mellitus, *CAD* coronary artery disease, *RA* rheumatic arthritis, *IFG* impaired fasting glucose

^a^the study by van Hoek et al. (2009) included two atorvastatin treatment arms with dosages of 10 and 80 mg/d respectively, and both the comparisons were included separately

^b^the study by Koh et al. (2010) included four atorvastatin treatment arms with dosages of 10, 20, 40, 80 mg/d respectively, and these comparisons were included separately

**Table 2:** Summary of study quality evaluated by Cochrane risk of biases tool

Author (year)	Sequence generation	Allocation concealment	Blinding of participants and personnel	Blinding of outcome assessment	Incomplete outcome data	Selective outcome reporting	Other potential threats	Total
Shetty 2004	Unclear	Unclear	Low	Low	Unclear	Unclear	Unclear	2
Koh 2005	Unclear	Unclear	Low	Low	Low	Unclear	Unclear	3
Chan 2008	Unclear	Unclear	Low	Low	Unclear	Unclear	Unclear	2
von Eynatten 2009	Unclear	Unclear	High	High	Low	Unclear	Unclear	1
van Hoek 2009–10 mg^a^	Unclear	Unclear	High	High	Low	Unclear	Unclear	1
van Hoek 2009–80 mg^a^	Unclear	Unclear	Low	Low	Low	Unclear	Unclear	3
Carnevale 2010	Low	Low	High	Low	Low	Unclear	Unclear	4
Koh 2010–10 mg^b^	Unclear	Low	Low	Low	Low	Unclear	Unclear	4
Koh 2010–20 mg^b^	Unclear	Low	Low	Low	Low	Unclear	Unclear	4
Koh 2010–40 mg^b^	Unclear	Low	Low	Low	Low	Unclear	Unclear	4
Koh 2010–80 mg^b^	Unclear	Low	Low	Low	Low	Unclear	Unclear	4
Koh 2011	Unclear	Unclear	High	High	Low	Unclear	Unclear	1
El-Barbary 2011	Unclear	Unclear	High	High	Low	Unclear	Unclear	1
Buldak 2012	Unclear	Unclear	High	High	Low	Unclear	Unclear	1

^a^the study by van Hoek et al. (2009) included two atorvastatin treatment arms with dosages of 10 and 80 mg/d respectively, and both the comparisons were included separately

^b^the study by Koh et al. (2010) included four atorvastatin treatment arms with dosages of 10, 20, 40, 80 mg/d respectively, and these comparisons were included separately

### Meta-analysis for the effect of atorvastatin on circulating adiponectin

**Fig. 2:**
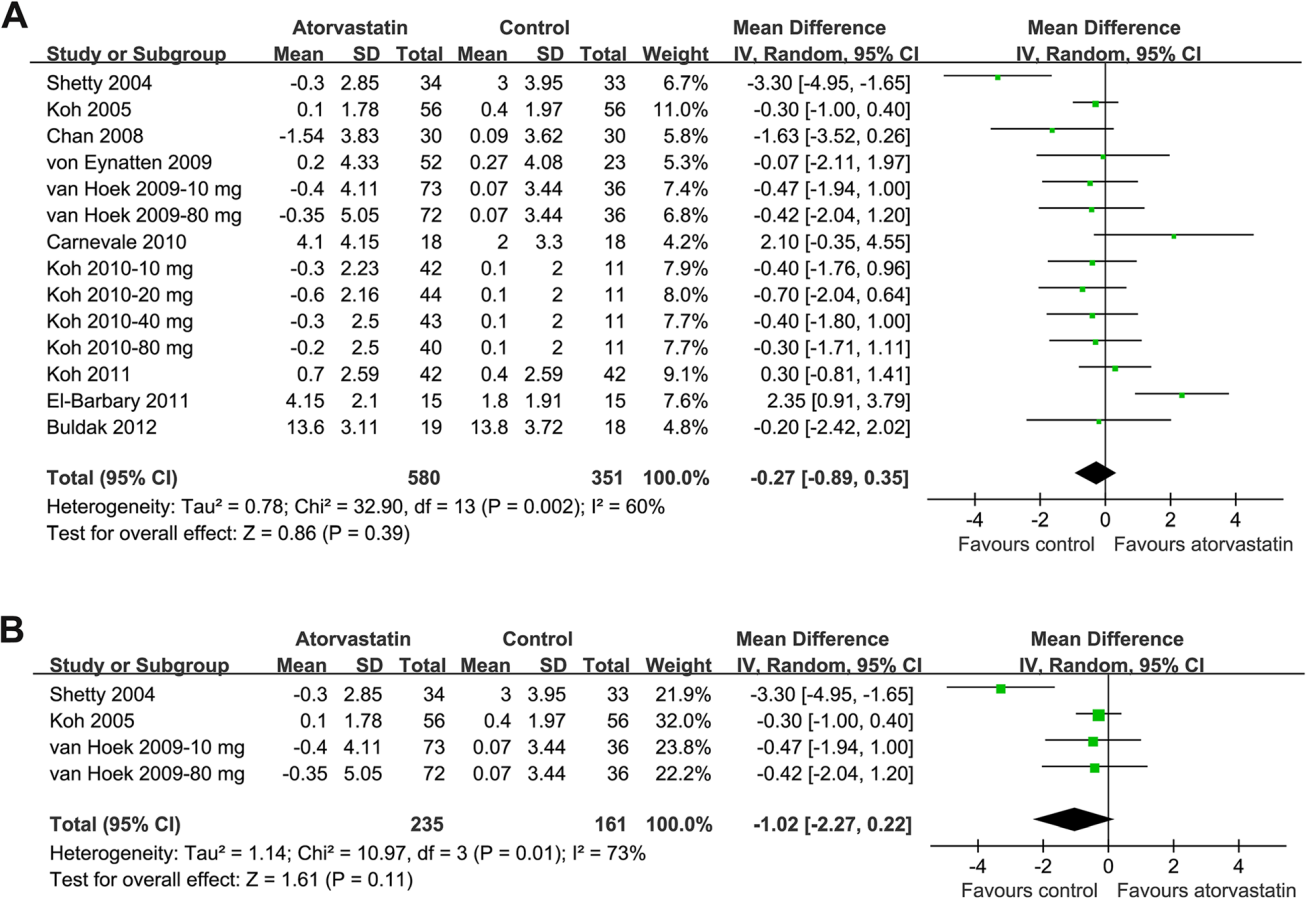
Forest plot for the meta-analysis of the effect of atorvastatin treatment on circulating adiponectin as compared with controls. **a**, forest plot for the overall meta-analysis; **b**, forest plot for the meta-analysis with double-blinded, placebo controlled RCTs

Fourteen datasets with 580 patients from the atorvastatin group and 351 patients from control group evaluated the influence of atorvastatin therapy on circulating adiponectin [18–27]. Significant heterogeneity was detected (p for Cochrane’s Q test = 0.002, I^2^ = 60%). Meta-analysis with a random-effect model showed that atorvastatin did not significantly affect circulating adiponectin as compared with controls (WMD = − 0.27 μg/mL, 95% CI: − 0.89 to 0.35 μg/mL, *p* = 0.39; Fig. 2a). Meta-analysis of studies limited to double-blinded and placebo-controlled RCTs showed similar results (WMD = − 1.02 μg/mL, 95% CI: − 2.27 to 0.22 μg/mL, *p* = 0.11; p for Cochrane’s Q test = 0.01, I^2^ = 73%; Fig. 2b).

### Meta-regression and subgroup analyses

Subsequently, univariate meta-regression and subgroup analyses were applied to evaluate whether predefined study characteristics have a significant influence on the outcome. Results of the univariate meta-regression analyses showed that among the predefined study characteristics including number of subjects, mean age, proportion of male patients, BMI, dose or treatment duration of atorvastatin, none of them was significant modifier of the outcome (p all > 0.05, Table 3). Moreover, subgroup analyses showed that atorvastatin treatment did not significantly affect the circulating adiponectin in studies stratified according to the above predefined study characteristics (p all > 0.05, Table 4).

**Table 3:** Impact of study characteristics to the effects of atorvastatin therapy on serum adiponectin concentrations: results of univariate meta-regression analyses

Study characteristics	WMD of serum adiponectin concentrations (ug/ml)		
	Coefficient	95% CI	p
Number of subjects	− 0.019	−0.043 to 0.005	0.10
Mean age (years)	−0.17	−0.44 to 0.10	0.35
Male (%)	−0.056	− 0.122 to 0.010	0.11
BMI (kg/m^2^)	−0.056	− 0.358 to 0.246	0.69
Dose (mg/d)	0.0038	−0.0218 to 0.0294	0.75
Duration (weeks)	−0.0023	−0.0734 to 0.0688	0.95

*Abbreviations*: *WMD* weighed mean difference, *CI* confidence interval, *BMI* body mass index

**Table 4:** Impact of study characteristics to the effects of atorvastatin therapy on serum adiponectin concentrations: results of subgroup analyses

Study characteristics	WMD of serum adiponectin concentration (ug/ml)				
	Comparisons (patients), n	I^2^	WMD [95% CI]	p^1^	p^2^
R, DB, PC studies					
Yes	4 (396)	73%	− 1.02 [−2.27, 0.22]	0.11	
No	10 (535)	50%	0.06 [−0.64, 0.77]	0.86	0.14
Crossover studies					
Yes	2 (196)	0%	−0.13 [− 0.72, 0.46]	0.66	
No	12 (735)	65%	−0.33 [−1.13, 0.47]	0.42	0.70
Patients with DM					
Yes	5 (396)	59%	−0.95 [−2.18, 0.29]	0.13	
No	9 (535)	57%	0.03 [−0.65, 0.71]	0.96	0.18
Patients with dyslipidemia					
Yes	7 (398)	0%	−0.28 [−0.75, 0.19]	0.24	
No	7 (533)	79%	−0.42 [−1.71, 0.87]	0.52	0.84
Mean age					
≤ 56 years	7 (419)	80%	0.02 [−1.13, 1.18]	0.97	
> 56 years	7 (512)	0%	−0.54 [−1.13, 0.04]	0.07	0.39
Male					
≤ 52%	8 (499)	56%	0.09 [−0.63, 0.81]	0.81	
> 52%	6 (432)	65%	−0.88 [−2.02, 0.27]	0.13	0.16
BMI					
≤ 26 kg/m^2^	9 (535)	57%	0.03 [−0.65, 0.71]	0.94	
> 26 kg/m^2^	5 (396)	59%	−0.95 [−2.18, 0.29]	0.13	0.18
Dose					
10 mg	6 (407)	12%	−0.32 [− 0.92, 0.28]	0.29	
20 mg	3 (206)	84%	−1.15 [−3.11, 0.81]	0.25	
40 mg	3 (159)	75%	0.67 [−1.18, 2.51]	0.84	
80 mg	2 (159)	0%	−0.35 [−1.42, 0.71]	0.52	0.62
Follow-up duration					
≤ 8 weeks	8 (520)	0%	−0.19 [− 0.62, 0.24]	0.40	
> 8 weeks	6 (411)	82%	−0.59 [−2.20, 1.02]	0.47	0.64
Adiponectin assay					
ELISA	11 (720)	38%	−0.01 [− 0.57, 0.54]	0.96	
RIA	3 (211)	85%	−1.48 [−3.74, 0.79]	0.20	0.22

*Abbreviations*: *WMD* weighed mean difference, *CI* confidence interval, *R* random, *DB* double-blinded, *PC* placebo-controlled, *BMI* body mass index, *ELISA* enzyme-linked immunosorbent assay, *RIA* radioimmunoassay

^1^*p* values for subgroup effects

^2^*p* values for subgroup interaction

### Publication bias

**Fig. 3:**
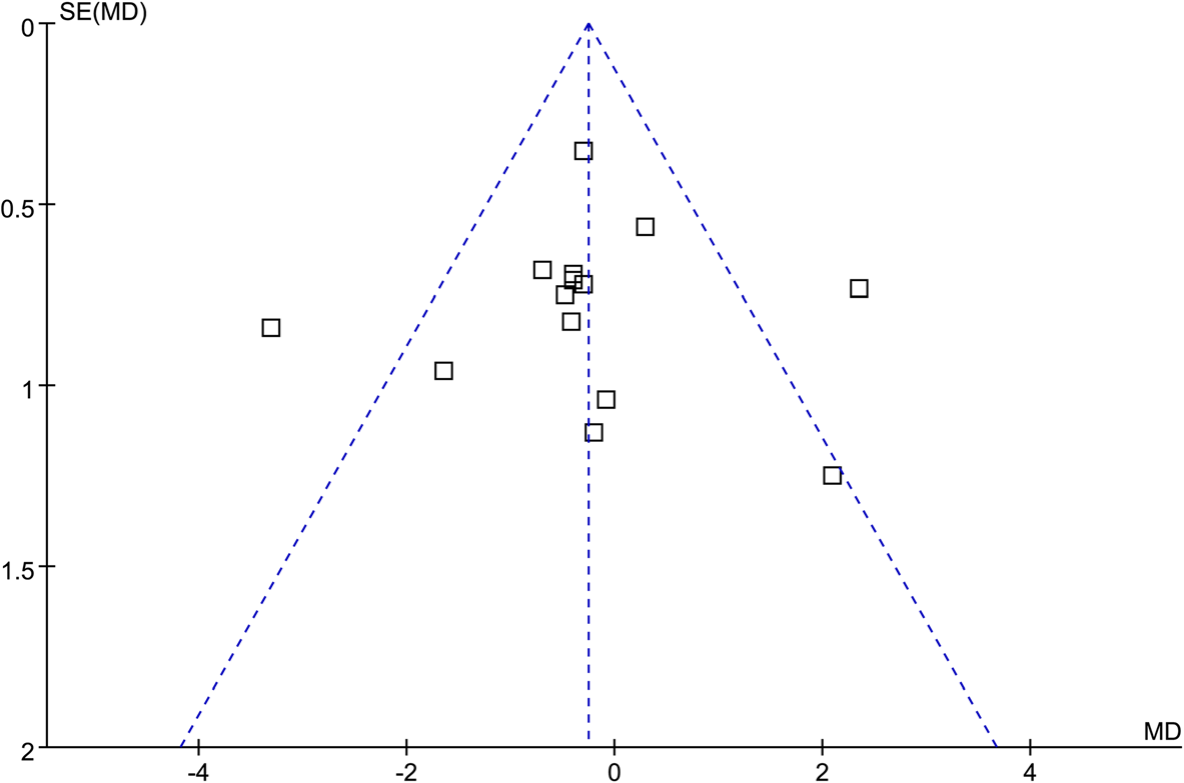
Funnel plots for the meta-analysis of the effect of atorvastatin treatment on circulating adiponectin

The forest plots for the meta-analysis of the effect of atorvastatin on circulating adiponectin were shown in Fig. 3. The plots were symmetrical on visual inspection, indicating low risk of publication bias. Egger’s regression test also demonstrated low risk of publication bias (*p* = 0.47).

## Discussion

In this meta-analysis, by pooling the results of fourteen datasets from ten RCTs, we found that treatment with atorvastatin was not associated with changes circulating adiponectin level compared to controls. Moreover, results of meta-regression and showed that study characteristics such as number of patients in each comparison, mean age, proportion of males, BMI, dose of atorvastatin, or treatment duration did not significantly affect the outcome. Subgroup analyses by stratification according to the characteristics in meta-regression as well as study design, disease status, and measurement methods for adiponectin consistently showed that atorvastatin did not affect adiponectin as compared to controls. Taken together, these results indicated that atorvastatin treatment does not significantly affect circulating adiponectin. Influences of atorvastatin on atherosclerosis and glycemic metabolism are not likely to be mediated by modulation of circulating adiponectin.

Results of our study showed that atorvastatin does not significantly affect circulating adiponectin as compared to controls. The results were consistent and independent of characteristics of study design, patient demographic factors, and dose and treatment durations of atorvastatin, indicating the robustness of the results. These findings demonstrated that the potential benefits of atorvastatin on cardiovascular and metabolic system are unlikely to be mediated via changing of circulating adiponectin. Similarly, in an experimental study of monosodium glutamate–treated obese mice, atorvastatin treatment reduced a series of inflammatory factors and maintained sensitivity of insulin without significant influence on circulating adiponectin [32]. Moreover, using angiotensin II treated adiponectin knockout mice, it was shown that atorvastatin attenuated myocardial fibrosis via adenosine monophosphate-activated protein kinase pathway but independent of the adiponectin signaling [33]. Taken together, modulation of circulating adiponectin may not be a principal mechanism underlying the benefits of atorvastatin on atherosclerosis and glycemic metabolism.

High-dose atorvastatin has been associated with more remarkable anti-inflammatory efficacy and cholesterol lowering action [34, 35]. However, results of our meta-regression and subgroup analyses did not support that dose of atorvastatin may affect its influence on circulating adiponectin. Moreover, since gender differences have been noticed for the circulating levels of adiponectin and its association with CV mortality [36, 37], it could be assumed that potential gender-difference may exist regarding the influence of atorvastatin on circulating adiponectin. Results of our meta-regression and subgroup analyses did not support the above assumption. However, since we do not have access to the individual patient data of each included RCT, a direct comparison between male and female participants was unable to perform, and our results were based on the data from study-level. Therefore, future studies are needed to determine whether atorvastatin could affect circulating adiponectin in a gender-dependent manner.

Our study has limitations. Firstly, the number of datasets included in the overall meta-analysis and subgroup analyses was limited. The results of meta-regression and subgroup analyses, therefore, should be cautiously interpreted. Large scale RCTs are needed to validate our findings. Secondly, the qualities of the included studies were generally moderate. Only three of them were double-blinded and placebo-controlled [18, 19, 21]. Well-designed high-quality RCTs are needed to confirm the results. Thirdly, patients with different disease status were included, which may cause the heterogeneity among the studies. Moreover, it has been reported that dietary factors, such as supplementation of omega-3 fatty acids may affect adiponectin level [38]. However, these factors were rarely controlled in the included studies. Finally, the treatment durations of the included studies were within 30 weeks. It remains to be determined whether atorvastatin could affect circulating adiponectin with treatment duration > 30 weeks.

## Conclusions

In conclusion, atorvastatin treatment does not significantly affect circulating adiponectin. Influences of atorvastatin on atherosclerosis and glycemic metabolism are not likely to be mediated by modulation of circulating adiponectin.

## Data Availability

All data generated or analyzed during this study are included in this article.

## Abbreviations

BMI: Mean body mass index
CI: Confidence intervals
CVD: Cardiovascular disease
DM: Diabetes mellitus
RCT: Randomized controlled trials
SD: Standard deviations
WMD: Weighted mean difference
